# Successful use of water-soluble contrast in patients with small bowel obstruction and virgin abdomen: A case report

**DOI:** 10.1016/j.ijscr.2021.01.004

**Published:** 2021-01-06

**Authors:** Sergio Mazzola Poli de Figueiredo, Sara Demola

**Affiliations:** Department of Surgery, University of Texas Medical Branch Hospital, 301 University Blvd, Galveston, TX 77555, United States

**Keywords:** Small bowel obstruction, Adhesions, Virgin abdomen, Water soluble contrast, Nonoperative management, Case report

## Abstract

**Introduction and importance:**

Nonoperative management of adhesive SBO is well established but remains a challenge in patients without prior abdominal surgery. We aim to report a case of successful nonoperative management with the use of enteral hypertonic water-soluble contrast administration in a patient with virgin abdomen.

**Case presentation:**

A healthy 24-year old man with no previous surgery presented to the emergency room with one day of abdominal pain. A CT abdomen and pelvis was consistent with SBO without clear anatomic etiology. The patient refused surgical intervention, so we performed a trial of nonoperative management. On hospital day 2, a repeat CT A/P with enterally administered water-soluble contrast showed resolution of SBO. The patient has had no symptoms since hospital discharge on 6 months follow up.

**Clinical discussion:**

Small bowel obstruction is most commonly secondary to adhesions from prior surgeries.

Even in patients with virgin abdomen, adhesions are the cause of SBO in 53%–73%.

Recent studies in patients with virgin abdomen showed that 92.1% that underwent nonoperative management did not have a recurrence of SBO with mean follow up of 4.5 years. The use of water-soluble contrast in patients with virgin abdomen was reported to have 92–97% success rate.

A meta-analysis showed a pooled prevalence of 7.7% of malignant etiology of SBO in these patients, more common with previous SBO admission or history of malignancy.

**Conclusion:**

Nonoperative management with the therapeutic use of hypertonic water-soluble contrast is a viable treatment option in select cases and avoids the morbidity of surgical exploration.

## Highlights

• Trial of nonoperative management is standard of care for adhesive SBO.
• Prior surgery is the most common cause of adhesions.
• Management of SBO in patients without prior surgery is a challenge.
• This is a case of SBO in a virgin abdomen patient successfully managed nonoperatively.

## 1 Introduction

Small bowel obstruction (SBO) is one of the most common causes for surgical admission and is responsible for more than 300,000 operations annually in the Unites states [1]. Adhesive bowel disease is the most common etiology in 70% of patients with small bowel obstruction. Prior intra-abdominal surgery is present in 80% of patients with adhesive SBO [2], while the remainder have prior peritonitis or no other clear cause for adhesions. Although nonoperative management is effective in 70–90% with adhesive small bowel obstruction [11], many large centers consider SBO in patient without prior abdominal surgery (virgin abdomen) an indication for operative intervention [3]. However, many recent studies have challenged this dogma suggesting that nonoperative management of small bowel obstruction in patients with virgin abdomen is an acceptable option for selected patients [[4], [5], [6], [7], [8], [9], [10]].

We aim to report a case of successful nonoperative management with the use of water-soluble contrast in a patient with virgin abdomen.

Informed patient consent was obtained for publication of the case details. All identifying information has been removed from this case report to protect patient privacy. This work has been reported in line with the SCARE 2020 criteria [12].

## 2 Case presentation

**Fig. 1:**
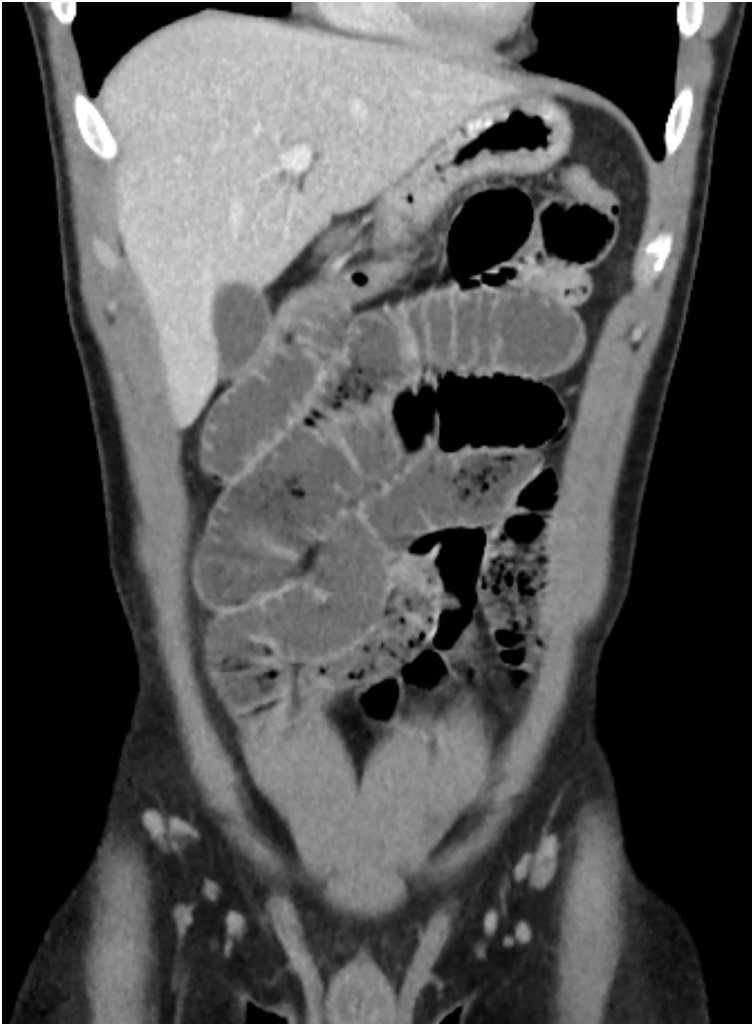
Coronal view of CT abdomen and pelvis on admission*.*

**Fig. 2:**
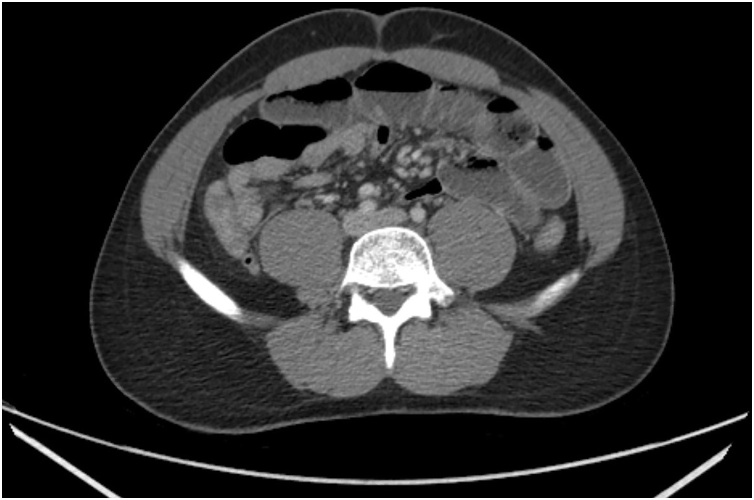
Axial view of CT abdomen and pelvis on admission.

**Fig. 3:**
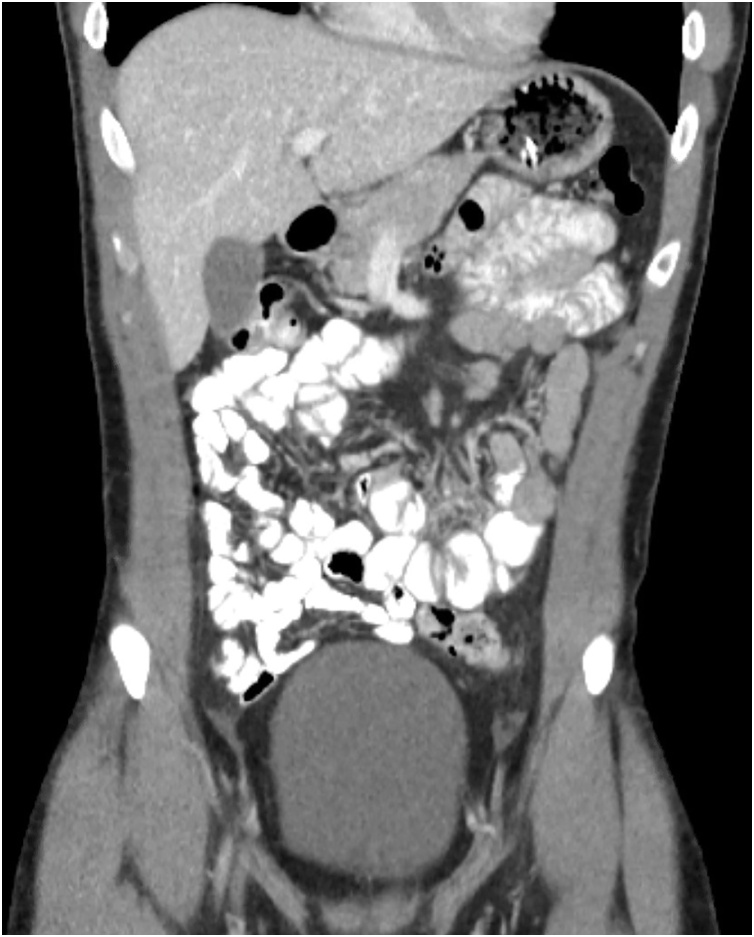
Coronal view of CT abdomen and pelvis with PO contrast after 24h of non-operative management of SBO*.*

**Fig. 4:**
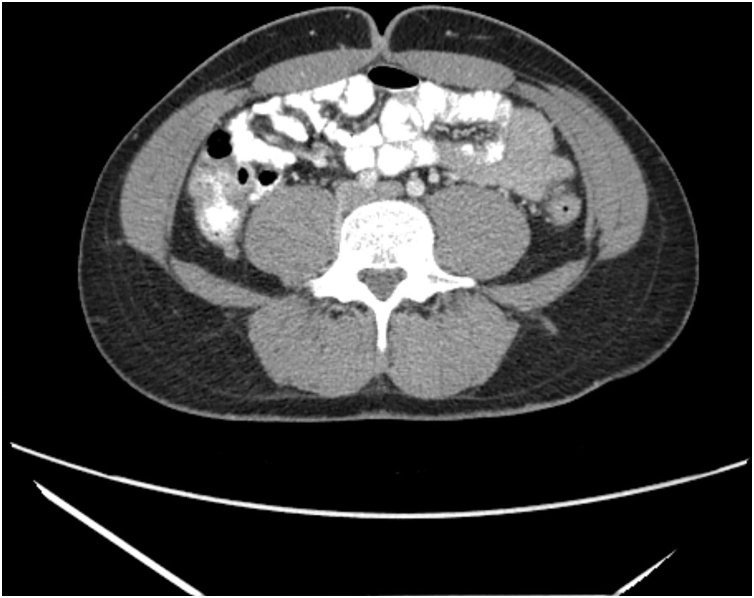
Axial view of CT abdomen and pelvis with PO contrast after 24h of non-operative management of SBO.

A healthy 24-year old male presented to the emergency room with one day history of sudden diffuse abdominal pain associated with nausea, vomiting and absence of bowel function. The patient had no past medical history, past surgical history, allergies or significant family history. On physical examination the patient had abdominal distention and tenderness to palpation diffusely but without peritonitis. No significant laboratory abnormalities were seen other than elevated hemoglobin (16.6). A CT abdomen and pelvis with IV contrast was obtained and showed multiple dilated small bowel loops with decompressed distal ileum and large bowel associated with small bowel feces sign (Fig. 1, Fig. 2). The patient was offered diagnostic laparoscopy given the setting of small bowel obstruction without clear etiology, but he refused any surgical intervention at this time. Decision was then made to perform a trial of non-operative management with intravenous hydration and nasogastric tube (NGT) decompression. Over the first 24 h of hospital admission, the patient had 1.4 L of output from NGT and Hemoglobin down trended to 13.8 after volume resuscitation. On hospital day 2, a repeat CT A/P with enterally administered water-soluble contrast showed no dilation of the small bowel and progression of contrast to the mid transverse colon (Fig. 3, Fig. 4). At that point the patient NGT was removed and diet was started. On hospital day 3 the patient was discharged given that he was tolerating a diet and had return of bowel function with resolution of abdominal pain. The patient had no symptoms since hospital discharge on 6 months follow up.

## 3 Discussion

Small bowel obstruction accounts for up to 15% of surgical admission for acute nontraumatic abdominal pain and is responsible for more than 300,000 operations annually in the United States [1,2]. Adhesive small bowel obstruction is the leading cause with up to 70% prevalence, but nonadhesive etiologies include incarcerated hernias, obstructive lesions (malignant or benign), volvulus and many other causes [1,11]. The prevalence of virgin abdomen in patient with SBO is 1.3%–9% [6,8,10]. Patients with virgin abdomen and no obvious cause of small bowel obstruction on imaging are commonly thought to have an occult malignant obstructive lesion that demands operative intervention for resolution [3]. Despite the classic dogma of early operative intervention in patients without previous abdominal surgery, many recent studies have shown that nonoperative management can be an acceptable option for some patients [[4], [5], [6], [7], [8], [9], [10]].

Even without previous abdominal surgery, adhesions were found to be the cause of SBO at time of operative intervention in 53.5%–73.5% [5,6]. Strajina et al. reported 44.8% of adhesions in patients that underwent therapeutic surgical exploration and 40% of negative surgical exploration in these patients [7].

Nonoperative management is effective in 70–90% of patients with SBO due to adhesive small bowel disease [11]. Recent studies showed that nonoperative management in patients with virgin abdomen also has a high success rate, most likely given that many of these patients will still have adhesive bowel disease despite having no prior abdominal surgery.

Tavangari et al. found in a study with 63 patients with virgin abdomen that 92.1% that underwent nonoperative management did not have a recurrence of SBO with mean follow up of 4.5 years [8], while Ng et al. reported 3.4% of SBO recurrences in 29 patients [5] and Strajina et al. reported 2 SBO recurrences (20%) in 10 patients that were managed nonoperatively with mean follow up of 22 months [7]. Nonoperative management with the use of water-soluble contrast in patients with virgin abdomen was reported to have 92–97% success rate in two studies with 36 and 38 patients [9,10].

An important disadvantage of nonoperative management is the risk of missing an underlying malignancy that was not suspected prior to surgical intervention. A recent metanalysis showed a pooled prevalence of 7.7% of malignant etiology of small bowel obstruction in patient with virgin abdomen, many of them not suspected prior to surgical intervention [4]. The same study also reported that presence of previous SBO admission and history of non-abdominopelvic malignancy were more likely to have malignant small bowel obstruction [4].

Given that our patient was young and did not have previous SBO or history of malignancy, he was successfully managed nonoperatively with the use of water-soluble contrast and persists asymptomatic 6 months after hospital discharge.

## 4 Conclusion

Adhesions are the most common cause of SBO in patients with virgin abdomen, just as in patients with prior surgery. Nonoperative management with the therapeutic use of hypertonic water-soluble contrast is a viable treatment option in select cases when adhesions are suspected and avoids the morbidity of surgical exploration.

## Declaration of Competing Interest

The authors report no declarations of interest.

## Sources of funding

None.

## Ethical approval

Case reports are exempt from the need of IRB approval in our institute.

## Consent

Written informed consent was obtained from the patient for publication of this case report and accompanying images. A copy of the written consent is available for review by the Editor-in-Chief of this journal on request.

## Author contribution

Conceptualization, Writing of manuscript, Literature review, Data Collection: Sergio Mazzola Poli de Figueiredo.

Data analysis and Reviewing of the final version of the manuscript: Sergio Mazzola Poli de Figueiredo, Sara Demola.

## Registration of research studies

N/A.

## Guarantor

Sergio Mazzola Poli de Figueiredo.

## Provenance and peer review

Not commissioned, externally peer-reviewed.
